# Supplementary material for: Identification of photoperiod-induced specific miRNAs in the adrenal glands of Sunite sheep (Ovis aries)
Source: Front Vet Sci. 2022 Jul 22;9:888207. doi: 10.3389/fvets.2022.888207 (PMC9354845; doi:10.3389/fvets.2022.888207)
Supplement: Supplementary file 1 [file Data_Sheet_1.ZIP › Supplementary file/miRDeep2 file.zip!/miRDeep2 file/Novel_Structure/LP42A1/Novel_618.pdf]

Provisional ID	:	NC_019460.2_8566
Score total	:	3.7
Score for star read(s)	:	3.9
Score for read counts	:	-3.9
Score for mfe	:	2.1
Score for randfold	:	1.6
Score for cons. seed	:	
Total read count	:	4
Mature read count	:	2
Loop read count	:	0
Star read count	:	2

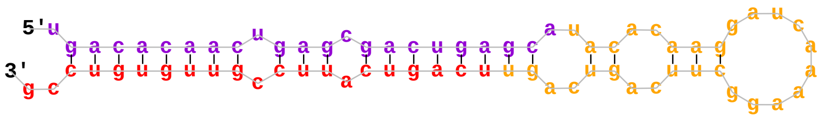

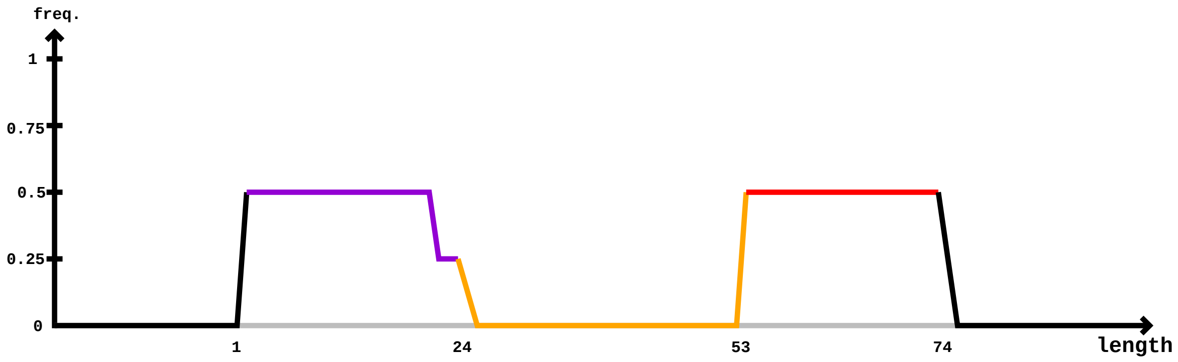


```
                 Star                                              Mature
5'- ccauuggguugcagagccugacacaacugagcgacugagcauacacaaggaucaaaaggcuucagucaguucagucauuccguuguguccgacucugugaccccauagacua   -3'    obs
    ccauuggguugcagagccugacacaacugagcgacugagcauacacaaggaucaaaaggcuucagucaguucagucauuccguuguguccgacucugugaccccauagacua          exp
    .....((((((((((.(.((((((((.(((.((((((((..((..(((..........)))..))..)))))))).))).)))))))).).))))))))))).........   reads    mm    sample
    ..................ugacacaacugagcgacuga..........................................................................   1        0     seq
    ..................ugacacaacugagcgacugagca.......................................................................   1        0     seq
    ......................................................................ucagucauuccguuguguccg.....................   1        0     seq
    ......................................................................ucagucauuccguuguguccga....................   1        0     seq
```
